# CD8^+^ T cells in neurodegeneration: friend or foe?

**DOI:** 10.1186/s13024-022-00563-7

**Published:** 2022-09-02

**Authors:** Dan Hu, Weiming Xia, Howard L. Weiner

**Affiliations:** 1 Ann Romney Center for Neurologic Diseases, Brigham and Women’s Hospital and Harvard Medical School, Boston, MA 02115 USA; 2 Geriatric Research Education and Clinical Center, Bedford VA Healthcare System, Bedford, MA 01730 USA; 3 Department of Pharmacology and Experimental Therapeutics, Boston University School of Medicine, Boston, MA 02118 USA

**Keywords:** CD8^+^ Tregs, T_EMRA_ cells, Neurodegeneration

## Main text

T cell infiltration is enhanced in disease-associated brain areas in neurodegenerative diseases such as multiple sclerosis (MS), Alzheimer’s disease (AD) and Parkinson’s disease (PD), with most of them being CD8^+^ T cells [1, 2]. Increased clonal expansion and heightened expression of T cell activation and cytotoxicity-associated genes in clonally expanded CD8^+^ T cells in the cerebrospinal fluid (CSF) are reported in patients with MS, AD or PD. [3–5] Clonal expansion of T cells indicates T cell recognition of a specific antigen and subsequent establishment of an immune response. CD8^+^ T cells are commonly viewed as pro-inflammatory cytotoxic T lymphocytes (CTLs) even though immune-suppressive CD8^+^ regulatory T cells (CD8^+^ Tregs) have been described for over a half centenary [6]. Therefore, it is understandable to conjecture that these disease-associated CD8^+^ T cells elicit immune responses and inflict cytotoxicity in the central nervous system (CNS) resulting in neurodegeneration [3, 4]. However, recent single-cell RNA sequencing (scRNA-seq) and single-cell T-cell receptor sequencing (scTCR-seq) analyses in conjunction with flow cytometric analysis reveal that both clonally expanded CD8^+^ T cells in neurodegenerative diseases [4, 5, 7] and immune suppressive CD8^+^ Tregs [8, 9] are terminally differentiated effector memory T cells (T_EMRA_) expressing high levels of cytotoxicity-associated molecules and sharing cell surface markers, raising the critical question of what role these clonally expanded CD8^+^ T cells play in neurodegenerative diseases. Here we discuss the phenotype and function of clonally expanded CD8^+^ T cells in neurodegenerative diseases and immune suppressive CD8^+^ Tregs and postulate their roles in neurodegeneration.

TCR Vβ repertoire analysis in MS patients shows that clonally expanded CD8^+^ T cells in MS lesions in the brain are reflected in peripheral blood and CSF, particularly, in CSF [10]. Therefore, analyzing clonally expanded CD8^+^ T cells in CSF, which is much more feasible than analyzing the sparse brain infiltrating T cells, is a valuable approach to study the role of T cells in neurodegenerative diseases. The recent advance in scTCR-seq and scRNA-seq techniques enables one to simultaneously measure TCR and gene expression profiles at single-cell resolution, which not only allows identifying clonally expanded CD8^+^ T cells in AD, PD and MS, but also reveals functional and physiological insights in these cells through analyzing corresponding global gene expression profiles [4, 5, 7]. CD45RA is a naïve T cell marker, but T_EMRA_ cells regain the expression of CD45RA while maintaining the CD27^−^CCR7^−^ cell surface marker characteristic of effector memory cells. Therefore, T_EMRA_ cells are conventionally defined as CD45RA^+^CD27^−^, CD45RA^+^CCR7^−^, or CD45RA^+^ CD27^−^CCR7^−^ T cells, which can be readily identified with flow cytometric analysis using fluorescent-conjugated antibodies specific for CD45RA, CD27 or CCR7. scRNA-seq analysis does not usually distinguish the RA and RO isoforms of CD45, and T_EMRA_ cells are defined as memory (CD27^−^CCR7^−^) T cells expressing high levels of T_EMRA_-associated genes such as *GZMA* (granzyme A), *GZMB* (granzyme B), *PRF1* (perforin) and *NKG7* [5]. T_EMRA_ cells are highly cytolytic but have a poor proliferation capacity. Flow cytometric analysis shows that circulating CD8^+^ T cells in MS tend to acquire a terminally differentiated phenotype [10], and CD8^+^ T_EMRA_ cells are also increased in peripheral blood and CSF in AD, and are negatively associated with cognition [4]. scRNA-seq and scTCR-seq analyses in conjunction with flow cytometric analysis of CSF cells reveal that clonally expanded CD8^+^ T cells from AD patients are CD45RA^+^CD27^−^CCR7^−/low^CD127^−^CD161^−^PD-1^−^ T_EMRA_ cells expressing high levels of granzyme genes, *NKG7*, *CST7*, *CCL4* and *CCL5* [4]. scRNA-seq and scTCR-seq analyses of CSF cells isolated from patients with PD show clonally expanded T cells were enriched in CD27^−^CCR7^−^GZMA^hi^GZMB^hi^PRF1^hi^NKG7^hi^ CD8^+^ T cells, and these cells also express high levels of *CCL5*, *CST7*, *GZMH* and *GZMK* [5]. scRNA-seq and scTCR-seq analyses show that clonally expanded CD8 T cells in MS express higher levels of CD8 effector function–related molecules including granzymes A and K, *NKG7*, *PFN1*, *CST7*, *CCL5*, and *CCL4* and express lower levels of *SELL* (CD62L), which are *CCR7*^−/low^*CD127*^−^ [7]. These gene expression characteristics indicate that, like in AD patients, clonally expanded CD8^+^ T cells in the CSF of patients with PD or MS are also T_EMRA_ cells. However, gene expression analysis does not reveal the cellular function of these cells. By consensus, CD8^+^ T_EMRA_ cells have high cytotoxicity and lyse target cells as a regular cytotoxic T lymphocyte (CTL) via T-cell receptor (TCR) recognition of a specific peptide presented by a compatible MHC molecule. Therefore, it is postulated that these clonally expanded CD8^+^ T_EMRA_ cells are highly pro-inflammatory and promote neurodegeneration [4].

Interestingly, the cell surface marker expression of clonally expanded CD8^+^ T_EMRA_ cells in the CSF of patients with AD is similar to that of CD161^−^CD56^+^ CD8^+^ Tregs identified in human peripheral blood a decade ago [11, 12]. Both are CD45RA^+^CD27^−^CCR7^−/low^CD127^−^CD161^−^PD-1^−^, though it is not specified whether these clonally expand CD8^+^ T_EMRA_ cells are CD56^+^. CD161^−^CD56^+^ CD8^+^ Tregs kill TCR-activated effector CD4^+^ T cells showing functional and cell surface marker similarities to the recently identified immune-suppressive KIR^+^CD8^+^ T cells, the human equivalent of mouse Ly49^+^CD8^+^ Tregs that prevent or dampen autoimmune responses [8, 13, 14]. Both CD161^−^CD56^+^ CD8^+^ Tregs and KIR^+^CD8^+^ T cells are CD45RA^+^CD27^−^CCR7^−^CD28^−^CD127^−^, and kill activated CD4^+^ T cells in a cell-cell contact-dependent manner. It has been reported that almost all KIR^+^ T cells are CD56^+^, and the majority of KIR^+^ T cells are CD8^+^ T cells [15]. These observations indicate that CD161^−^CD56^+^ CD8^+^ Tregs and immune-suppressive KIR^+^CD8^+^ T cells are likely the same immune regulatory CD8^+^ T cell subpopulation. Since CD161^−^CD56^+^ CD8^+^ Tregs and KIR^+^CD8^+^ T cells are CD45RA^+^CD27^−^CCR7^−^, they should be considered as CD8^+^ T_EMRA_ cells following the conventional classification. Moreover, scRNA-seq and scTCR-seq analyses show that, like clonally expanded CD8^+^ T cells in neurologic diseases, clonally expanded KIR^+^CD8^+^ T cells express elevated levels of *GZMH*, *GZMB*, and *PRF1* [8]. Increased KIR^+^CD8^+^ T cells are found in the peripheral blood and inflamed tissues of patients with autoimmune diseases including MS and during viral infection. However, KIR^+^CD8^+^ T cells do not seem to be induced to aggravate the autoimmunity. Instead, KIR^+^CD8^+^ T cells are shown to specifically kill activated pathogenic or autoreactive CD4^+^ T cells acting as immune-suppressive regulatory T cells [8]. Moreover, unlike conventional T_EMRA_ cells that are terminally differentiated with poor proliferative capacity, CD161^−^CD56^+^ CD8^+^ Tregs proliferate robustly and maintain their functional characteristics after long-term culturing [11, 12]. Clearly, CD161^−^CD56^+^ CD8^+^ Tregs and KIR^+^CD8^+^ T cells do not fit into the conventional concept of CD8^+^ T_EMRA_ cells. Thus, the key question is whether clonally expanded CD8^+^ T_EMRA_ cells in AD, PD, or MS are immune-suppressive regulatory cells that kill pathogenic CD4^+^ T cells, or they are proinflammatory cytolytic cells that fuel neurodegeneration.

**Fig. 1:**
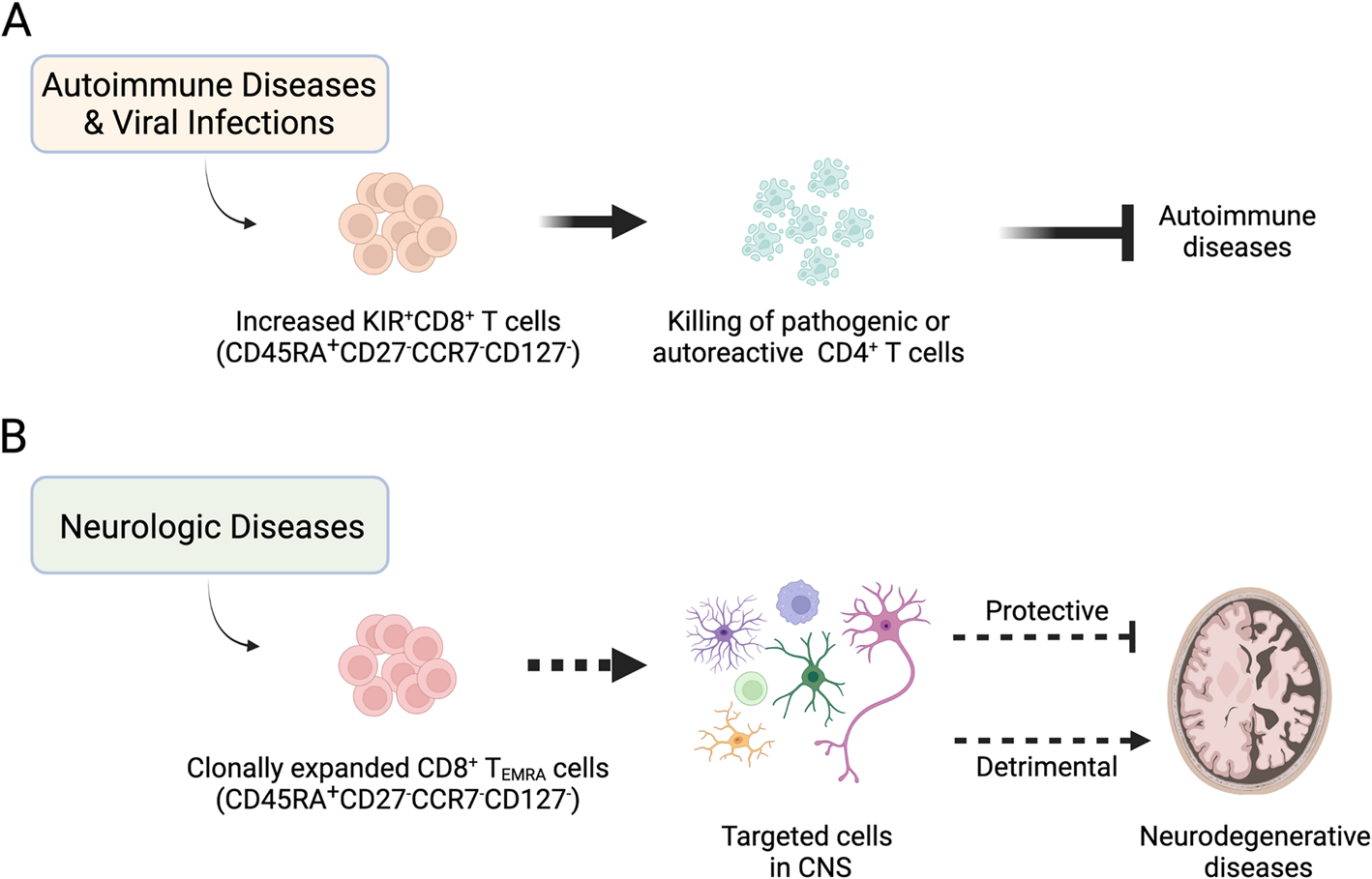
CD45RA^+^CD27^−^CCR7^−^CD127^−^ CD8^+^ T_EMRA_ cells in human diseases. **A** In human autoimmune diseases such as multiple sclerosis and during viral infection as such COVID-19, CD45RA^+^CD27^−^CCR7^−^CD127^−^ KIR^+^CD8^+^ T cells are increased in the peripheral blood and inflamed tissues of patients. KIR^+^CD8^+^ T cells kill T-cell receptor activated pathogenic and autoreactive CD4^+^ T cells to prevent the development of autoimmune diseases and dampen autoimmune immune responses. **B** CD45RA^+^CD27^−^CCR7^−^CD127^−^ CD8^+^ T_EMRA_ cells are clonally expanded in the cerebrospinal fluid from patients with neurodegenerative diseases such as multiple sclerosis (an autoimmune disease) and Alzheimer’s disease. The function and specific cell-type classification of these CD8^+^ T_EMRA_ cells are unknown. It is yet to be determined if they act like cytotoxic T lymphocytes to damage the center nervous system, or regulatory T cells to subdue rogue immune responses. Created with BioRender (Biorender.com)

The categorization of T_EMRA_ cells is more a developmental stage classification than a cell lineage/type definition. Unlike CD4^+^ T cells that have well-defined transcription factors, cell surface markers and cytokines to categorize the population into subtypes such as Th1, Th2, Th17, Tfh, Tregs, etc., the sub-classification of CD8^+^ T lineages is still vague. With currently available gene expression information, it cannot be determined whether these clonally expanded CD8^+^ T_EMRA_ cells and CD8^+^ Tregs are completely or partially overlapping or non-overlapping populations. Theoretically, CD8^+^ T_EMRA_ cells in disease-impacted brain areas could be either CTLs that promote detrimental immune responses or regulatory cells that are induced by the undesired immune responses in the brain to dampen the detrimental immune responses, or a mixture of both cell types. The shared cell type-defining surface markers of terminally differentiated CTLs and immune-suppressive CD8^+^ Tregs signify the necessity for new CD8^+^ T cell classification markers. Studies dedicated to identifying the diversity of CD8^+^ T_EMRA_ cells and each subpopulation’s biological function are needed to fill in a blank spot in immunology. Interestingly, single-cell trajectory analysis shows that the terminal effector CD8^+^ T cells (T_EMRA_ cells) in the CSF from PD patients display two differentiation directions, with one expressing high levels of killer-like receptors (KLRs) and killer cell immunoglobulin-like receptors (KIRs) [5], raising the possibility of that some of the clonally expanded CD8^+^ T cells in neurologic diseases are immune suppressive KIR^+^CD8^+^ T cells (Fig. 1). Similar analysis can also be carried out with the single cell multi-omic datasets on CSF cells from patients with AD or MS to explore the diversity of CD8^+^ T_EMRA_ cells. CD161^−^CD56^+^ CD8^+^ Tregs were identified based on functional characterization of cloned CD8^+^ T cells [11, 12]. Cloning of CD8^+^ T_EMRA_ cells from the CSF followed by functional analysis in conjunction with global transcriptomic analysis may be a practical approach to define their diversity and unique molecular markers, and to understand their roles in neurodegeneration.

## Data Availability

Not applicable. The study contains publicly available data from published studies.
